# Editorial: New strategies for analysis and evaluation of pharmaceuticals and natural products

**DOI:** 10.3389/fchem.2023.1212346

**Published:** 2023-05-04

**Authors:** Linnan Li

**Affiliations:** Institute of Chinese Materia Medica, Shanghai University of Traditional Chinese Medicine, Shanghai, China

**Keywords:** analysis, evaluation, pharmaceuticals, natural products, editorial

It is with great pleasure that we present this Research Topic on “New Strategies for Analysis and Evaluation of Pharmaceuticals and Natural Products” in the Analytical Chemistry section of Frontiers in Chemistry. As guest editor, I have had the privilege of working with esteemed co-editors, Dr. Li Yang, Dr. Jian-Bo Wan, and Dr. Jiaqi Fu, and we are grateful to the contributing authors for their excellent research articles.

The Research Topic comprises six articles, including two review articles and four original research articles, covering a range of Research Topic including traditional Chinese medicine, metabolomics, chemometrics, and nanotechnology. The review articles provide valuable insights into the current state of research and future directions in the fields of traditional Chinese medicine and mass spectrometry-based metabolomics. The original research articles showcase novel analytical techniques and approaches to evaluate the therapeutic potential of natural products and their mechanisms of action.

In the Research Topic, Zhang et al. reviews Epimedii Herba, an ancient Chinese herbal medicine used for rheumatoid arthritis, and provides a comprehensive overview of its potential therapeutic benefits. Liu et al. investigates the effects of Buyang Huanwu decoction on gut microbiota and lipid metabolism in a rat model of co-morbid type 2 diabetes mellitus and obesity. Yu et al. characterizes carotenoids in Lycium barbarum fruit and their 5α-reductase inhibitory activity, using an innovative approach combining deep eutectic solvents extraction with UPC2-PDA-Q-TOF-MSE. Cui et al. presents the identification of chemosensitizing agents of colorectal cancer in Rauvolfia vomitoria using an NMR-based chemometric approach. Huang et al. demonstrates the potential of nanotechnology in developing sensitive and selective analytical methods for the detection of neurotransmitters, focusing on the sensitive detection of noradrenaline in human whole blood. Guo et al.’s review article discusses mass spectrometry-based metabolomics for discovering active ingredients and exploring the action mechanisms of herbal medicine, highlighting the potential of metabolomics in the analysis and evaluation of natural products.

This Research Topic provides a glimpse into the latest developments in the field of analytical chemistry for the analysis and evaluation of pharmaceuticals and natural products. We hope that the knowledge will serve as a valuable resource for researchers and professionals working in this field, and inspire new ideas and approaches for the analysis and evaluation of these important classes of compounds. We also would like to thank all the authors for their excellent contributions and the reviewers for their valuable feedback and support.

